# Dietary Strawberries Improve Biomarkers of Antioxidant Status and Endothelial Function in Adults with Cardiometabolic Risks in a Randomized Controlled Crossover Trial

**DOI:** 10.3390/antiox10111730

**Published:** 2021-10-29

**Authors:** Arpita Basu, Kenneth Izuora, Nancy M. Betts, Jeffrey L. Ebersole, Robert Hal Scofield

**Affiliations:** 1 Department of Kinesiology and Nutrition Sciences, University of Nevada at Las Vegas, Las Vegas, NV 89154, USA; 2 Section of Endocrinology, University of Nevada School of Medicine at Las Vegas, Las Vegas, NV 89154, USA; kenneth.izuora@unlv.edu; 3 Department of Nutritional Sciences, Oklahoma State University, Stillwater, OK 74078, USA; nancy.betts@okstate.edu; 4 School of Dental Medicine, University of Nevada at Las Vegas, Las Vegas, NV 89154, USA; jeffrey.ebersole@unlv.edu; 5 Section of Endocrinology and Diabetes, University of Oklahoma Health Sciences Center, Oklahoma City, OK 73104, USA; Hal-Scofield@omrf.org; 6 Arthritis and Clinical Immunology, Oklahoma Medical Research Foundation, Oklahoma City, OK 73104, USA

**Keywords:** strawberries, cardiometabolic, superoxide dismutase, antioxidant status, adhesion molecules

## Abstract

Strawberries, a popularly consumed berry fruit, are rich in bioactive compounds with antioxidant effects. In this study, we examined the effects of two dietary achievable doses of strawberries on the antioxidant status and biomarkers of endothelial function in adults with features of metabolic syndrome and a confirmed low baseline of fruit and vegetable intake. In a 14-week randomized controlled crossover study, participants were assigned to one of three groups for four weeks separated by a one-week washout period: control powder, one serving (low dose: 13 g strawberry powder/day), or 2.5 servings (high dose: 32 g strawberry powder/day). Blood samples and health data were collected at baseline and at the end of each four-week phase of intervention. Thirty-three participants completed all three phases of the trial. Significant increases were observed in serum antioxidant capacity and superoxide dismutase activity as well as decreases in lipid peroxidation after both low and high dose strawberry phases when compared with the control phase. Significant decreases were also observed in soluble vascular cell adhesion molecule-1 and tumor necrosis factor-α with the high dose strawberry phase. These data confirm that consuming strawberries for four weeks significantly improves antioxidant status, endothelial function, and inflammation in adults with cardiometabolic risks.

## 1. Introduction

In recent years, accumulated evidence from several lines of research has supported the benefits of dietary berries for improving cardiometabolic risk in the adult population [1,2,3,4]. Most of these benefits have been attributed to the role of berry phytochemicals, especially the polyphenolic flavonoids and other bioactive compounds, towards minimizing oxidative stress, inflammation, and related pathological pathways underlying diabetes and vascular dysfunction [5]. Oxidative stress has been positively correlated with endothelial dysfunction [6,7], and biomarkers of endothelial dysfunction, such as adhesion and inflammation molecules, have been shown to be modulated by dietary polyphenols found in plant-based diets [8]. While epidemiological studies show an inverse association of dietary berry consumption with diabetes risk [1,2], randomized controlled trials on the effects of berries on biomarkers of antioxidant/oxidative stress status and endothelial function are lacking in adults with cardiometabolic risk factors.

Antioxidant enzymes and their cofactors, such as the concentration of glutathione, have been shown to be decreased in erythrocytes of patients with type 2 diabetes (T2D), consistent with increased susceptibility to oxidative damage and other complications [9,10]. The enzymes of the glutathione system, such as glutathione reductase and glutathione peroxidase, are also altered in diabetic vs. non-diabetic control subjects [11,12]. Among the categories of dietary phytochemicals with known antioxidant effects in humans, anthocyanins and ellagic acid present in the berries have been shown to improve antioxidant enzyme profiles. In a clinical trial in healthy volunteers (*n* = 57), consumption of anthocyanin-rich fruit juice caused a significant increase in superoxide dismutase (SOD) activity when compared to the placebo group [13]. In another study among patients with T2D (*n* = 44), ellagic acid supplementation significantly increased activities of SOD, as well as glutathione peroxidase enzymes, and increased total blood antioxidant capacity vs. the placebo group [14]. Anthocyanins and ellagic acid are the major bioactive compounds in dietary berries, especially in strawberries [15], which justifies the opportunity to examine their effects on these antioxidant biomarkers. While these results have been encouraging, the small number of such reported trials, as well as the lack of such studies conducted in adults with metabolic syndrome or prediabetes, makes it difficult to clarify the potential role of berries and their bioactive compounds on antioxidant profiles in these adults at risk. Endothelial dysfunction underlies the initiation and progression of atherosclerotic cardiovascular disease [16]. Again, very few studies have examined the role of dietary berries on biomarkers of endothelial dysfunction. In one clinical study among adults with hypercholesterolemia (*n* = 150), administration of purified anthocyanins significantly decreased adhesion molecules and biomarkers of inflammation vs. placebo [17]. On the other hand, in adults with cardiometabolic risk (*n* = 18), consumption of a wild blueberry drink decreased DNA oxidative damage but did not affect biomarkers of endothelial function and inflammation [18]. As conflicting results could arise from differences in sample size and participants’ metabolic characteristics, there is a need to examine these effects in controlled studies in adults with metabolic syndrome.

Among dietary berries, commonly consumed berries such as blueberries, cranberries, and strawberries, have shown distinct benefits in adults with metabolic syndrome [19]. Our group has previously reported the effects of functional foods such as green tea, blueberries, and strawberries on improving antioxidant profiles and biomarkers of endothelial function in adults with diabetes risk [20,21,22]. We recently reported a dose–response study in which we observed improved insulin resistance following intake of a dietary achievable dose of strawberries for four weeks in adults with obesity and above optimal blood LDL-cholesterol [23]. We are now reporting serum biomarkers of antioxidants, endothelial function, and inflammation in the same study population. We hypothesize that dietary supplementation of strawberries will improve these biomarkers in adults at risk for developing T2D and related CVD pathologies.

## 2. Methods

### 2.1. Study Design and Criteria

As previously described, this was a randomized, crossover-controlled, double-blind study for 14 weeks in which adult participants were recruited at three different campuses at the University of Nevada at Las Vegas, University of Oklahoma Health Sciences Center, and the Oklahoma State University [23]. The study was approved by the respective Institutional Review Boards (IRB number 1119274) and was registered at http://clinicaltrials.gov/. (Identifier: NCT03441620). All participants provided written informed consent. The study criteria, as previously explained, involved inclusion based on above optimal serum LDL-cholesterol and features of metabolic syndrome. Participants were excluded if they took medications for lowering lipids and glucose levels, were allergic to strawberries, and/or had chronic and acute health conditions that required medical treatment [23].

### 2.2. Intervention and Protocol

Participants were randomized into one of three interventions for four weeks in the crossover design with a one-week washout phase between each phase: control, one serving of strawberries, or two-and-half servings of strawberries. The strawberries were administered as freeze-dried strawberry powder (FSP). The caloric content of the control and strawberry powders ranged from 122 to 124 kcal, and the carbohydrate content ranged from 27 to 29 g daily. While the control powder did not provide bioactive compounds, the total polyphenols in one and two-and-half servings of FSP were 400 and 960 mg, respectively, and total anthocyanin content was 38 and 92 mg, respectively, as a daily dose. In addition, fiber intake ranged from 3 to 5 g for the control and strawberry interventions [23]. The participants were instructed to reconstitute the powders in plain drinking water and consume them as a beverage twice a day and at least six to eight hours apart. Health history data and blood samples were collected at baseline and at the end of each four-week phase of the study.

### 2.3. Biomarkers of Antioxidant Status

Serum samples collected at baseline and at the end of each four-week phase of the study were analyzed for antioxidant biomarkers. Serum catalase and glutathione peroxidase (GPX) were determined using a spectrophotometric assay based on the manufacturer’s protocol (Oxis Research, Portland, OR, USA). Glutathione reductase (GR) and superoxide dismutase (SOD) activity were measured using commercially available kits (Cayman Chemical, Ann Arbor, MI, USA). Reduced glutathione was measured as previously described by Beutler et al. [24] based on the absorbance of the yellow thiolate anion at 412 nm. Total serum antioxidant capacity was measured using the metmyoglobin assay developed by Miller et al. [25]. Serum nitrite was measured using the Griess Reagent System (Promega Corporation, Madison, WI, USA). Serum malondialdehyde (MDA) levels were measured by the method of Jain et al. [26] based on the reaction of MDA with thiobarbituric acid to produce a complex that can be determined spectrophotometrically at 532 nm. All the inter-assay CVs ranged between 3 and 7%.

### 2.4. Biomarkers of Endothelial Function and Inflammation

Serum adhesion molecules (soluble E-selectin [sE-Selectin], soluble P-selectin [sP-Selectin], soluble intercellular adhesion molecule-1 [sICAM-1], and soluble vascular cell adhesion molecule-1 [sVCAM-1]), as well as inflammatory analytes (interleukin [IL]-6, IL-1β, tumor necrosis factor-α), were measured using a Quantikine human immunoassay kits (R&D Systems, Minneapolis, MN, USA). All these inter-assay CVs ranged between 4 and 8%.

### 2.5. Statistical Analysis

Continuous variables are expressed as means ± SD and no value was detected as an outlier. We used a mixed model ANOVA to examine the main effects of treatment, time, and interactions, which were then used to analyze differences in outcomes at the end of each four-week phase of intervention. Baseline values were included as covariates for each outcome variable. Outcomes were modeled as repeated measures with the subject as a random effect and with unstructured variance for treatment/time. The sequence of intervention was included in all models to test for carry-over effects, and none were detected. As an exploratory analysis, we also used correlation methods to examine positive or inverse correlations among biomarkers of antioxidant status and inflammation. All *p* values were 2-tailed and main effects and interaction effects were considered if <0.05. Analyses were performed using SPSS, version 26.0 (SPSS, Armonk, NY, USA).

## 3. Results

As previously reported, a total of 33 adults (31 females, 2 males) completed all phases of the randomized crossover study [23]. Enrolled participants had a baseline BMI of 33 ± 3 kg/m^2^ (mean ± SD), 2 of the 33 were males who had a low intake of fruits and vegetables at baseline (1.0 ± 0.4, and 1.3 ± 0.2 cups/day, respectively), and were insulin resistant based on HOMA-IR (3.6 ± 1.5). Overall, BMI did not change after any phase of the interventions in the study.

As shown in Table 1, among the biomarkers of antioxidant status, serum SOD and antioxidant capacity significantly increased after the 4-week low dose as well as the high dose, strawberry phase when compared to baseline and control phases. Serum MDA significantly decreased after the 4-week low dose and high dose strawberry phases when compared to the baseline and control phases. Serum levels of glutathione showed a borderline significant increase following only the high dose strawberry phase.

As shown in Table 2, among the biomarkers of endothelial dysfunction and inflammation, both serum sVCAM-1 and TNF-α were significantly decreased after the 4-week high dose strawberry phase when compared to baseline, control, and low dose strawberry phases.

Finally, in our exploratory analyses as shown in Table 3, we observed some significant correlations among biomarkers in a model adjusted for baseline age and BMI. As expected, we observed a significant inverse correlation between glutathione and antioxidant capacity with sVCAM-1, and between SOD and TNF-α. In contrast, we observed a significant positive correlation between serum MDA, a biomarker of lipid oxidation, and TNF-α, a biomarker of inflammation.

## 4. Discussion

We observed some notable differences in antioxidant status and biomarkers of endothelial dysfunction and inflammation following a dietary intervention of two different doses of strawberries in adults with cardiometabolic risk in a randomized crossover study. There was a significant improvement in antioxidant status reflected by an increase in serum SOD and overall antioxidant capacity, and a concomitant decrease in lipid peroxidation following a four-week supplementation of one serving as well as two-and-a-half servings, of strawberries daily. Decreases were also observed in serum levels of the adhesion molecule, sVCAM-1, and inflammatory biomarker, TNFα, following the same dose of strawberries. To our knowledge, this is the first clinical study to identify improvements in antioxidant status following a short-term supplementation of whole strawberries in adults with habitual low consumption of fruits and vegetables, obesity, and insulin resistance.

Our findings of improved antioxidant status conform to the few reports on the role of dietary berries conducted mostly in healthy adults. Strawberry supplementation has been shown to increase plasma antioxidant capacity in healthy humans, though none of these studies reported effects on antioxidant enzymes in the participants [27,28,29]. However, a variety of other dietary bioactive rich compounds, such as curcumin and anthocyanins, have also been shown to increase antioxidant enzymes in clinical trials. In adults with metabolic syndrome, curcuminoid-piperine supplementation significantly increased serum SOD activity as well as decreased serum MDA levels [30]. Similar results showed decreases in MDA and serum SOD activity following red wine supplementation in healthy volunteers [31]. In a meta-analysis of 17 RCTs involving grape polyphenol supplementation, significant increases in antioxidant capacity and SOD activity were observed in healthy volunteers. Interestingly, no differences were noted in other antioxidant enzyme markers [32]. In another study of healthy humans, a 4-week supplementation of acai berry juice significantly increased total blood antioxidant capacity, as well as catalase and GPX activities [33]. Thus, while fruit-derived polyphenols may differentially affect each of the antioxidant enzyme as biomarkers of improved health, there is a clear gap in the knowledge of their effects in adults with cardiometabolic risks that our findings have addressed.

Our findings of decreased serum in the vascular adhesion molecule, VCAM-1, and inflammatory molecule, TNF-α, following the higher dose supplementation of strawberries in adults with cardiometabolic risks agree with our previously reported studies. These include the effects of consuming approximately four-and-half servings of strawberries for eight weeks that decreased serum VCAM-1 in adults with metabolic syndrome [34] and the effects of a similar dose of strawberries that decreased serum TNF in adults with knee osteoarthritis and cardiometabolic risk [35]. The current findings have additional clinical application as improvements were observed even at a lower dose of strawberry intake than supplements in our earlier studies. This contrasts with the administration of wild blueberry juice for seven days in adults at risk for T2D that showed no changes in glycemic control and biomarkers of inflammation or adhesion molecules [36]. In preclinical models of diet-induced atherosclerosis in mice, a combination of freeze-dried fruit and vegetable powder decreased serum TNF-α at a dose of approximating eight to nine servings/day in humans [37]. Similar decreases in inflammatory biomarkers, including interleukins and TNFα, as well as adhesion molecules, were observed in endothelial cells treated with Aronia berry extract [38]. Furthermore, Pietro et al. reported a comprehensive review of dietary polyphenols by themselves or in combination with carotenoids, as found in fruits and vegetables, to significantly reduce the expression of biomarkers of endothelial dysfunction [39]. While these combined results provide some mechanistic insight into our clinical observations, there remains an urgent need for further studies to document these effects of dietary berries in adults with prediabetes and at risk for atherosclerotic CVD. In this exploratory analysis, we observed significant inverse correlations of biomarkers of antioxidant status with VCAM-1 and TNFα, as well as a positive correlation between lipid peroxidation and TNFα in our pooled sample of all participants. Similar correlations have been reported in observational studies in adults with metabolic syndrome or T2D [40,41,42], thereby confirming the potential impact of antioxidant interventions derived from whole foods on these populations at risk for CVD.

Some limitations of this study include it being a randomized crossover study and that each phase lasted only four weeks. Longer duration studies should provide more robust outcomes. We specifically included adults not taking medications for chronic conditions such as T2D and CVD. Because of this, there are some limitations on the generalizability to the larger population since a high number of people are medicated for these conditions. Only MDA was explored as a biomarker of oxidative stress byproducts; future studies should include effects on protein oxidation products and their associated effects on the vasculature. Despite these limitations, we have reported novel data on the role of dietary achievable strawberry bioactive agents in improving systemic antioxidant status and inflammation in adults with cardiometabolic risks.

## 5. Conclusions

Our findings support that the dietary consumption of whole strawberries, fresh or frozen, as a source of antioxidants, can perform as active nutritional therapy for CVD and diabetes prevention and management. In addition to improving antioxidant status, strawberries also lowered the selected biomarkers of inflammation and endothelial dysfunction. These data further strengthen the evidence of the cardioprotective role of strawberries in adults with obesity and elevated cholesterol.

## Figures and Tables

**Table 1:** Serum antioxidants and oxidative stress biomarkers in adults with the metabolic syndrome following control, low, and high-dose strawberry powder for a period of four weeks each in a randomized crossover study.

Variable	Baseline	Control (4-Week)	Strawberry (LD) (4-Week)	Strawberry (HD) (4-Week)	*p*-Value ^1^ (Treatment)
Serum catalase, U/mL ^2^	55 ± 14	64 ± 11	74 ± 17	67 ± 9	0.25
Serum glutathione, µM	1021 ± 420	997 ± 312	1121 ± 332	1143 ± 412	0.05
Serum GR, nmol/min/mL	56 ± 14	52 ± 16	62 ± 18	63 ± 12	0.28
Serum GPX, mU/mL	22 ± 11	25 ± 9	33 ± 8	29 ± 10	0.34
Serum SOD, U/mL	0.02 ± 0.01 ^a^	0.03 ± 0.02 ^a^	0.04 ± 0.02 ^b^	0.06 ± 0.04 ^b^	**0.02**
Serum nitrite, µM	32 ± 19	26 ± 8	23 ± 11	25 ± 15	0.28
Serum antioxidant capacity, µmol/L	5.2 ± 4.3 ^a^	4.9 ± 4.2 ^a^	6.5 ± 4.5 ^b^	7.3 ± 3.5 ^b^	**0.02**
Serum MDA, nmol/mL	5.16 ± 2.06 ^b^	4.86 ± 3.21 ^a^	3.50 ± 2.06 ^b^	3.21 ± 1.31 ^b^	**0.002**

Data presented as means ± SD. N = 33/group. HD = High dose (~2.5 servings of strawberries/day); LD = low dose (~1.0 serving of strawberries/day). GR = glutathione reductase; GPX = glutathione peroxidase; MDA = malondialdehyde; SOD = superoxide dismutase. ^1^ P for main effect of treatment from a mixed model ANOVA. Repeated measures adjusted for baseline values. ^a,b^ Different superscripts indicate significantly different values. *p* < 0.05 in bold font. ^2^ P for significant time effect with higher values at 14 weeks compared to baseline and four weeks (*p* < 0.05).

**Table 2:** Serum biomarkers of endothelial dysfunction and inflammation in adults with the metabolic syndrome following control, low, and high-dose strawberries for a period of four weeks each in a randomized crossover study.

Variable	Baseline	Control (4-Week)	Strawberry (LD) (4-Week)	Strawberry (HD) (4-Week)	*p*-Value ^1^ (Treatment)
Serum sICAM-1, ng/mL	321 ± 109	333 ± 113	305 ± 95	311 ± 99	0.34
Serum sVCAM-1, ng/mL	288 ± 114 ^a^	279 ± 103 ^a^	262 ± 102 ^a^	231 ± 98 ^b^	**0.02**
Serum sP-selectin, ng/mL	118 ± 65	124 ± 77	108 ± 75	111 ± 55	0.26
Serum sE-selectin, ng/mL	37.4 ± 12.5	32.1 ± 10.5	28.6 ± 9.5	31.6 ± 11.5	0.21
Serum IL-6, pg/mL	7.6 ± 4.4	6.8 ± 5.1	7.2 ± 6.4	6.3 ± 3.4	0.17
Serum IL-1β, pg/mL	12.7 ± 5.8	11.4 ± 6.4	10.7 ± 5.3	13.4 ± 6.5	0.19
Serum TNF-α, pg/mL	6.3 ± 3.5 ^a^	5.7 ± 4.5 ^a^	4.5 ± 3.2 ^a^	3.8 ± 3.6 ^b^	**0.01**

Data presented as means ± SD. N = 33/group. HD = High dose (~2.5 servings of strawberries/day); LD = low dose (~1.0 serving of strawberries/day); IL-6 = interleukin-6; IL-1β = interleuin-1beta; sICAM-1 = soluble intercellular adhesion molecule-1; sVCAM-1 = soluble vascular cell adhesion molecule-1; TNF-α = tumor necrosis factor alpha. ^1^ P for main effect of treatment from a mixed model ANOVA. Repeated measures adjusted for baseline values. ^a,b^ Different superscripts indicate significantly different values. *p* < 0.05 in bold font.

**Table 3:** Partial correlation coefficients among serum antioxidants, lipid oxidation, and inflammatory biomarkers adjusted for age and BMI at baseline and at the end of the study.

Variable by Time	sICAM-1	sVCAM-1	IL-1β	TNF-α
Glutathione Baseline End	0.21 0.15	**−0.42** **−0.35**	0.14 0.08	−0.12 −0.09
SOD Baseline End	−0.05 −0.06	−0.18 −0.23	−0.02 −0.02	**−0.23** **−0.19**
Antioxidant capacity Baseline End	0.08 0.11	**−0.25** **−0.32**	−0.05 −0.05	−0.03 −0.02
MDA Baseline End	0.07 0.05	0.13 0.15	0.03 0.02	**0.28** **0.35**

N = 33 at baseline and end of study. Abbreviations: BMI = body mass index; MDA = malondialdehyde; SOD = superoxide dismutase; IL−1β = interleuin-1beta; sICAM-1 = soluble intercellular adhesion molecule-1; sVCAM-1 = soluble vascular cell adhesion molecule-1; TNF-α = tumor necrosis factor alpha. *p* < 0.05 in bold font.

## Data Availability

The datasets analyzed in the current study are not publicly available due to ethical reasons and because our participants only gave their consent for the use of their data by the original team of investigators.

## Clinical Trial Registry

Clinicaltrials.gov identifier NCT03441620.
